# NSAIDs Modulate *CDKN2A, TP53,* and DNA Content Risk for Progression to Esophageal Adenocarcinoma

**DOI:** 10.1371/journal.pmed.0040067

**Published:** 2007-02-27

**Authors:** Patricia C Galipeau, Xiaohong Li, Patricia L Blount, Carlo C Maley, Carissa A Sanchez, Robert D Odze, Kamran Ayub, Peter S Rabinovitch, Thomas L Vaughan, Brian J Reid

**Affiliations:** 1 Human Biology Division, Fred Hutchinson Cancer Research Center, Seattle, Washington, United States of America; 2 Public Health Sciences Division, Fred Hutchinson Cancer Research Center, Seattle, Washington, United States of America; 3 Department of Medicine, University of Washington, Seattle, Washington, United States of America; 4 Cellular and Molecular Oncogenesis Program, Wistar Institute, Philadelphia, Pennsylvania, United States of America; 5 Department of Pathology, Harvard Medical School and Brigham and Women's Hospital, Boston, Massachusetts, United States of America; 6 Department of Gastroenterology, Virginia Mason Medical Center, Seattle, Washington, United States of America; 7 Department of Pathology, University of Washington, Seattle, Washington, United States of America; 8 Department of Epidemiology, University of Washington, Seattle, Washington, United States of America; 9 Genome Sciences, University of Washington, Seattle, Washington, United States of America; Genome Institute of Singapore, Singapore

## Abstract

**Background:**

Somatic genetic *CDKN2A, TP53,* and DNA content abnormalities are common in many human cancers and their precursors, including esophageal adenocarcinoma (EA) and Barrett's esophagus (BE), conditions for which aspirin and other nonsteroidal anti-inflammatory drugs (NSAIDs) have been proposed as possible chemopreventive agents; however, little is known about the ability of a biomarker panel to predict progression to cancer nor how NSAID use may modulate progression. We aimed to evaluate somatic genetic abnormalities with NSAIDs as predictors of EA in a prospective cohort study of patients with BE.

**Methods and Findings:**

Esophageal biopsies from 243 patients with BE were evaluated at baseline for *TP53* and *CDKN2A (p16)* alterations, tetraploidy, and aneuploidy using sequencing; loss of heterozygosity (LOH); methylation-specific PCR; and flow cytometry. At 10 y, all abnormalities, except *CDKN2A* mutation and methylation, contributed to EA risk significantly by univariate analysis, ranging from 17p LOH (relative risk [RR] = 10.6; 95% confidence interval [CI] 5.2–21.3, *p* < 0.001) to 9p LOH (RR = 2.6; 95% CI 1.1–6.0, *p* = 0.03). A panel of abnormalities including 17p LOH, DNA content tetraploidy and aneuploidy, and 9p LOH was the best predictor of EA (RR = 38.7; 95% CI 10.8–138.5, *p* < 0.001). Patients with no baseline abnormality had a 12% 10-y cumulative EA incidence, whereas patients with 17p LOH, DNA content abnormalities, and 9p LOH had at least a 79.1% 10-y EA incidence. In patients with zero, one, two, or three baseline panel abnormalities, there was a significant trend toward EA risk reduction among NSAID users compared to nonusers (*p* = 0.01). The strongest protective effect was seen in participants with multiple genetic abnormalities, with NSAID nonusers having an observed 10-y EA risk of 79%, compared to 30% for NSAID users (*p* < 0.001).

**Conclusions:**

A combination of 17p LOH, 9p LOH, and DNA content abnormalities provided better EA risk prediction than any single *TP53, CDKN2A,* or DNA content lesion alone. NSAIDs are associated with reduced EA risk, especially in patients with multiple high-risk molecular abnormalities.

## Abstract 2

In a ten-year study of people with Barrett's esophagus, nonsteroidal anti-inflamatory drugs were associated with reduced risk of esophageal adenocarcinoma, especially in patients with multiple high-risk molecular abnormalities.

## Editors' Summary

**Background.:**

Normally, the cells in the human body divide only when extra cells are needed, after an injury, for example. Sometimes, however, cells accumulate genetic changes (mutations) that allow them to divide uncontrollably to form a disorganized mass or tumor. If these altered cells also acquire mutations that allow them to spread around the body, a malignant tumor or cancer results. Scientists have identified numerous genetic changes that occur in tumors and are now investigating whether these molecular abnormalities can be used as “biomarkers” to choose the best treatments for patients, to identify who will benefit from cancer-prevention strategies, to detect cancer early, and to predict which cancers are most likely to become life-threatening. This last application is particularly important for cancers with a well-defined premalignant stage. Because the cells in premalignant tissues have acquired some of the genetic changes required for cancer development, they are more likely to become malignant than normal cells. Barrett's esophagus, for example, is a premalignant disorder of the muscular tube that takes food from the mouth to the stomach. People with Barrett's esophagus are much more likely to develop esophageal cancer than the general population.

**Why Was This Study Done?:**

Esophageal cancer is often incurable by the time it is detected, so it would be helpful to know which people with Barrett's esophagus are most likely to develop esophageal cancer—only 1 in 200 of them develop cancer each year. In this study, the researchers evaluated whether a panel of genetic alterations could identify this subset of patients. They also investigated whether the regular use of aspirin or other nonsteroidal anti-inflammatory drugs (NSAIDs) affects the risk of developing esophageal cancer in people with Barrett's esophagus—other evidence suggests that NSAIDs may help to prevent several types of cancer, including esophageal cancer.

**What Did the Researchers Do and Find?:**

The researchers took esophageal tissue samples from patients with Barrett's esophagus and looked for alterations in the genes encoding the tumor-suppressor proteins TP53 and CDKN2A. These proteins normally stop cells dividing but are often inactivated in cancer cells by mutation of one of the two gene copies that encode each of them and also loss of the other copy (so-called “loss of heterozygosity” or LOH). The researchers also looked for changes in the cellular DNA content of the samples (tumor cells often contain unusual amounts of DNA) and asked the study participants about their NSAID use before waiting to see which participants developed esophageal cancer. After 10 y, the participants whose tissue samples had LOH of the short arms (p) of Chromosome 17 or 9 (the sites of the genes encoding TP53 and CDKN2A, respectively), or an altered DNA content, were more likely to have developed esophageal cancer than those without these abnormalities; those whose samples contained all three abnormalities had the highest risk of developing esophageal cancer. Overall, just 12% of patients with no abnormalities but nearly 80% of patients with three abnormalities developed esophageal cancer. NSAID use reduced the risk of cancer development in all the participants, but its effect was greatest in those with three genetic abnormalities.

**What Do These Findings Mean?:**

These findings suggest that the combined measurement of 17pLOH, 9pLOH, and cellular DNA content might be a powerful way to identify those patients with Barrett's esophagus who are most likely to develop esophageal cancer. They also suggest that NSAID use is associated with a reduced risk of esophageal cancer, particularly in patients with multiple genetic abnormalities. Because very few participants developed cancer during the study, these results need confirming in more patients. Also, the ability of NSAIDs to prevent the progression of Barrett's esophagus to esophageal cancer needs testing in multicenter randomized trials; the use of the panel of abnormalities described here to identify the people with Barrett's esophagus most at risk of developing esophageal cancer should facilitate such studies.

**Additional Information.:**

Please access these Web sites via the online version of this summary at http://dx.doi.org/10.1371/journal.pmed.0040067.

- CancerQuest information from Emory University (Atlanta, Georgia, United States) on cancer biology, including the role of tumor suppressor proteins
- US National Cancer Institute patient and physician information on esophageal cancer and its prevention
- MedlinePlus encyclopedia pages on Barrett's esophagus and esophageal cancer
- Cancerbackup (UK charity) patient information on esophageal cancer and Barrett's esophagus

## Introduction

Rapid advances in understanding the molecular pathogenesis of neoplasia have raised the possibility that molecular abnormalities may be used as “biomarkers” for cancer risk stratification and early detection as well as possible entry criteria for cancer-prevention trials [1–4]. Identification of inherited, highly penetrant mutations in some cancer-susceptibility genes is being incorporated into clinical practice as well as cancer-prevention strategies for patients with many familial cancer syndromes, including inherited breast cancer, hereditary nonpolyposis colon cancer, and adenomatous polyposis coli [5–10]. Although progress in developing predictive biomarkers from common somatic genetic abnormalities in at-risk tissues has been less striking, there is some evidence that this approach may be successful. Based on a genetic progression model for head and neck cancer [11,12], a series of retrospective, longitudinal studies have been performed on patients with the premalignant condition oral leukoplakia, resulting in potential biomarker panels for risk stratification [13–16]. Some of these biomarkers have been proposed as entry criteria for a randomized cancer-prevention trial using cyclo-oxygenase-2 and epidermal growth factor receptor inhibitors [4]. Understanding how modifiable exposures interact with the somatic genetic composition of a neoplasm will be important for individualized interventions and cancer prevention.

Barrett's esophagus (BE) is a premalignant condition that predisposes to esophageal adenocarcinoma (EA). The incidence of this cancer has risen dramatically in the United States, Western Europe, Australia, and other developed countries over the past three decades, with little sign of abating [17,18]. Unfortunately, EA is typically lethal unless detected early, having an overall survival rate of only 13.7% [19]. Endoscopic surveillance is recommended for early detection in BE [20], and there has been recent interest in the potential for cancer prevention using nonsteroidal anti-inflammatory drugs (NSAIDs) based on data from case control and cohort studies [21–25], as well as preclinical models [26,27]. Analysis of genetic progression of BE has identified abnormalities in the tumor-suppressor genes *TP53* and *CDKN2A,* as well as DNA content abnormalities (tetraploidy and aneuploidy) as critical events in the evolution of EA [28–40]. Several prospective studies have suggested that individual somatic genetic abnormalities derived from this progression model may identify those patients with BE who are at increased risk for progression to EA, but no study has evaluated the combined contributions of genetic abnormalities for EA risk prediction [34,41–45].

Using data collected prospectively over a 10-y period from a long-standing cohort study of patients with BE, we investigated the role of host factors in combination with somatic genetic abnormalities in the development of EA. The aims of the study were to determine whether somatic genetic abnormalities involving *TP53, CDKN2A,* and DNA content were predictors of progression to EA and whether regular NSAID use modulates risk of these abnormalities for future EA.

## Methods

### Study Design

This was a prospective, longitudinal study of the Seattle Barrett's Esophagus Study cohort. It constituted a phase 4 study according to the National Cancer Institute's Early Detection Research Network classification [3]. The Seattle Barrett's Esophagus Study was approved by the Human Subjects Division of the University of Washington in 1983 and was renewed annually thereafter with reciprocity from the Institutional Review Board of the Fred Hutchinson Cancer Research Center from 1993 to 2001. Since 2001, the study has been approved annually by the Institutional Review Board of the Fred Hutchinson Cancer Research Center with reciprocity from the Human Subjects Division of the University of Washington.

### Participants

Two hundred and seventy-four participants were eligible as defined by a diagnosis of metaplastic columnar epithelium with intestinal metaplasia in esophageal biopsies, the absence of esophageal malignancy at or prior to baseline endoscopy, and having had at least one follow-up endoscopy. Two hundred and forty-three participants had sufficient tissue for flow cytometry, mutation, and LOH analysis in the same DNA samples and represented the final study cohort. Study participants entered surveillance from 1983 to 1999 (Table 1). The baseline endoscopy was defined as the first endoscopy from 5 January 1995 to 2 December 1999. Since this is a long-standing cohort, we assessed the enrollment-time effect by performing statistical analyses with all patients, and also with the subset of 211 patients, excluding the 32 patients who entered the cohort before 1990, and found no change in the conclusions of the study. This study was conducted at a specialty research and referral center, and thus our cohort is considered a high-risk patient population. We included all cancers that developed subsequent to the baseline evaluation so that accurate risk-stratification models could be developed based on findings at a single baseline endoscopy [44].

**Table 1:**
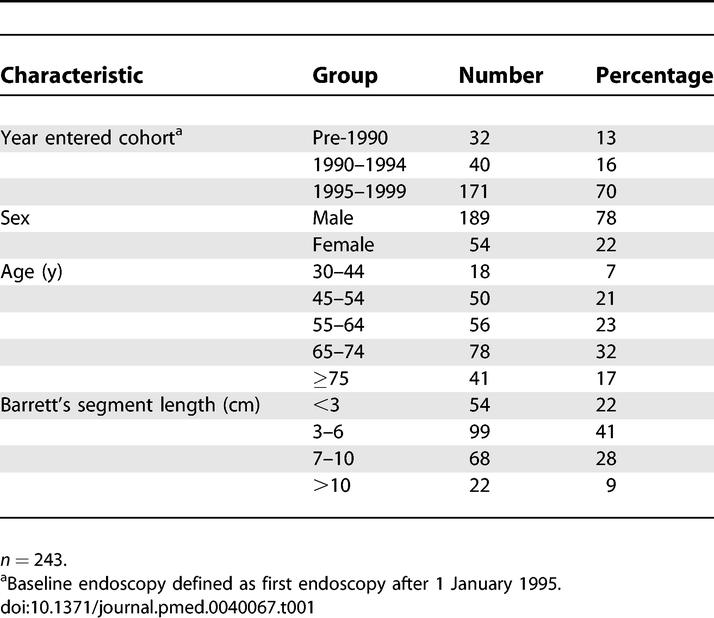
Cohort Characteristics

All research participants were counseled concerning the risks and benefits of endsocopic biopsy surveillance for BE. Patients with high-grade dysplasia (HGD) were also counseled concerning the risks and benefits of esophagectomy and endoscopic therapies. Endoscopic biopsy protocols used in the Seattle Barrett's Esophagus Study have been published previously [46]. Briefly, all patients had one biopsy evaluated for biomarkers every 2 cm in the Barrett's segment regardless of histologic diagnosis. In patients without HGD, four-quadrant biopsies were obtained for histologic evaluation every 2 cm in the Barrett's segment. If, after fully informed consent, participants with HGD opted for endoscopic biopsy surveillance reserving intervention for cancer if detected, they were evaluated by an intensive protocol of four-quadrant biopsies every 1 cm in the Barrett's epithelium at closely timed intervals for 4 mo, after which time endoscopies were typically repeated approximately every 6 mo [47]. Exclusion of patients who were diagnosed with EA that developed within 4 mo of their baseline procedure (*n* = 4) did not alter the conclusions of the study. Participants with maximum baseline diagnosis of HGD had an average of 7.3 endoscopies (median = 6.5, range 2–18), compared to those with less HGD at baseline, who had an average of 4.8 endoscopies (median = 4, range 2–20).

The cohort is typical for gender, age, and Barrett's segment length compared to other specialty research centers (Table 1) [48–51]. The cohort included 189 males and 54 females with a mean age of 62 y (median = 64 y, range 30–87 y) at entry into the study. This cohort was comprised of participants with maximum baseline histologic diagnoses including negative for dysplasia (*n* = 67), indefinite (*n* = 78), low-grade dysplasia (*n* = 48), and HGD (*n* = 50) using previously published pathology criteria [43,52]. The mean Barrett's segment length, defined by total centimeters between the ora serrata and the distal end of the tubular esophagus proximal to the flattening of the gastric folds, was 5.6 cm (median = 5 cm, range 0–20 cm). Participants were followed for a total of 17,139 patient-months with a mean of 71 mo (median = 80.5 mo, range 2.3–130.8 mo).

### Endoscopy and Biopsy

Endoscopy and biopsy were performed using a standard protocol [43]. Participants with a history of HGD (*n* = 50) had histologic evaluation of four-quadrant biopsies at 1-cm intervals in the Barrett's segment, whereas those without a history of HGD (*n* = 193) had histologic analysis of four-quadrant biopsies at 2-cm intervals. Histologic analyses were evaluated in biopsies obtained at the baseline evaluation and at all follow-up endoscopies, whereas *TP53, CDKN2A,* and DNA content analyses were evaluated at a single baseline endoscopy. For each baseline endoscopy, samples were characterized for 17p LOH spanning *TP53, TP53* mutations, DNA content abnormalities including tetraploidy and aneuploidy, 9p LOH spanning the *CDKN2A (p16)* locus, *CDKN2A* promoter methylation, and *CDKN2A* mutation in one biopsy every 2 cm in the Barrett's segment (average = 3.2, range 1–11 biopsies analyzed per study participant). For a participant to be counted as having a molecular abnormality at baseline, the abnormality must have been detected in more than one flow-purified DNA fraction or in a single sample that has been confirmed in a second, independent reaction.

### Flow Cytometry

Biopsies were processed by DNA content (DAPI) and Ki67/DNA content flow cytometry to determine 4N fraction and ploidy, and to purify proliferating cells using previously validated techniques [43,45,53]. Flow-purified fractions are defined as Ki67-positive (proliferating) 2N, 4N, increased 4N/tetraploid (cells with DNA content between 3.85 and 4.10N that comprise >6% of the total cells), and aneuploid populations (distinct DNA content peak representing >2.5% of cells). Cell-cycle analysis was performed on 1,569 flow-purified fractions with a median of 6.5 (range 1–29) cell-cycle fractions depending on Barrett's segment length.

### DNA Extraction, Whole-Genome Amplification and LOH Analysis

DNA was extracted from flow-purified cell populations using either standard phenol/chloroform or the Puregene DNA Isolation Kit as recommended by the manufacturer (Gentra Systems, Minneapolis, Minnesota, United States). Whole-genome amplification using primer extension preamplification was performed with each sorted fraction and three constitutive controls per participant [53]. LOH data was obtained from 1,331 and 1,284 flow-purified fractions at 17p and 9p loci, respectively, as detailed previously [34,44,53]. Thirteen microsatellite loci were evaluated, including the 17p loci D17S1298 (3.87 Mbp), D17S1537 (6.10 Mbp), *TP53*-ALU (AAAAT)*_n_* in intron 1 (7.77 Mbp), *TP53* (CA)*_n_* (7.77 Mbp), D17S786 (9.01 Mbp), D17S974 (10.72 Mbp), D17S1303 (11.06 Mbp), and Chromosome 9p loci D9S2169 (5.19 Mbp), D9S935 (5.19 Mbp), D9S925 (18.28 Mbp), D9S932 (24.43 Mbp), D9S1121 (25.39 Mbp), and D9S1118 (31.92 Mbp). Physical map locations were determined from the University of California, Santa Cruz (Santa Cruz, California, United States) version hg16 July 2003 assembly (http://genome.cse.ucsc.edu). LOH was defined as Q^LOH^ ≤ 0.4 or ≥ 2.5 for loss which spanned *TP53* or *CDKN2A* [34,44,53]. For convenience, we used the nomenclature of 17p LOH and 9p LOH to describe LOH events spanning these genes. LOH was not scored for rare telomeric or centomeric loss events that did not span *TP53* or *CDKN2A.* 17p LOH for all participants and 9p LOH in HGD patients have been previously published [34,44].

### *CDKN2A* Promoter Methylation Analysis

Genomic DNA from flow-purified Barrett's epithelium was evaluated for *CDKN2A* promoter methylation in 175 flow-purified fractions from 121 participants. Only a subset (*n* = 121) of the patients had *CDKN2A* methylation tested because the bisulfite treatment assays used required large amounts of DNA, and the DNA from our flow-purified fractions was insufficient for all molecular assays to be performed. This patient subset was representative of the entire 243-patient cohort with no statistically significant difference in follow-up time, sex, age, segment length, and cancer outcome between the methylation-assayed group and the non-assayed group. DNA was bisulfite treated and methylation-specific PCR was performed with modifications as detailed previously [31,54,55]. Human genomic DNA treated in vitro with Sss I methyltransferase (New England Biolabs, Beverly, Massachusetts, United States) was used as the methylated control. In a subset of cases, promoter methylation was determined and/or verified by directly sequencing PCR products of bisulfite-treated genomic DNA using published primers and methods [31]. Methylation data from a subset of patients has been previously reported [31].

### DNA Sequencing

Genomic or primer extension preamplification DNA was sequenced using either BigDye or BigDyeV3 Terminator cycle sequencing (Applied Biosystems, Foster City, California, United States) on either an ABI 377, ABI 3730, or ABI 3700 DNA sequencer. Wild-type sequences for each participant were confirmed using constitutive samples. All mutations were confirmed by at least two independent PCR and sequencing reactions and, in cases of ambiguity, by direct sequencing of genomic DNA. Evaluation of mutation of exons 5–9 of the *TP53* gene was performed on 1,118 flow-purified fractions under conditions described previously [56]. *TP53* mutations from patients with HGD were reported by Prevo et al. [56], and additional new *TP53* mutations not previously reported are available from the corresponding author (PCG). Mutation analysis of exon 2 of the *CDKN2A* gene was performed in 1,109 flow-purified fractions, detailed by Paulson 2006 (unpublished data) and with methods described previously [31].

### Use of Aspirin and Other NSAIDs

Questionnaires and methods for determining host variables were as described previously [25] and were available for 241 participants. Use of aspirin and other NSAIDs in the current study was defined in the same way as NSAID use, including NSAID use in follow-up, as described in Vaughan et al. [25]. Briefly, duration and frequency of regular aspirin and NSAID use were assessed, with “regular” defined by use at least once per week for ≥6 mo. The NSAID variable took into account changes in aspirin and NSAID medication use during the follow-up period. It has been shown previously that there are no significant differences between the protective associations of aspirin and other NSAIDs in this cohort [25]. Patients were classified as being users (including patients who were regular aspirin or other NSAID users within 1 y of the baseline interview or at any time during follow-up, *n* = 157), or nonusers (former users or those who had never used NSAIDs, *n* = 84).

### Statistical Analysis

**Figure 1:**
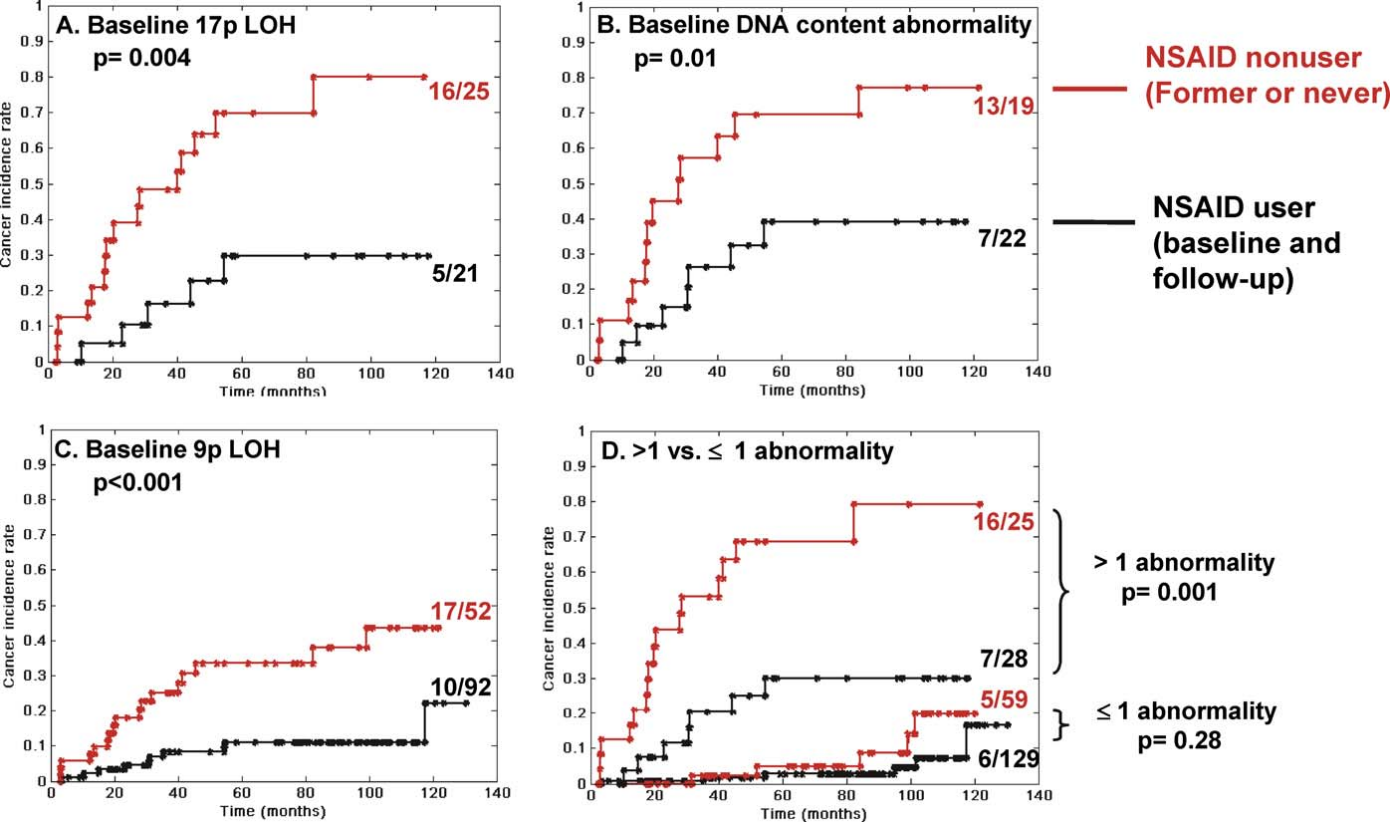
Modulation of EA Risk by NSAIDs in Participants with Different Baseline Abnormalities Two hundred and forty-one patients are classified according to whether they have (A) baseline 17p LOH (*n* = 46), (B) baseline DNA content abnormalities (aneuploidy and/or tetraploidy) (*n* = 41), (C) baseline 9p LOH (*n* = 144), or (D) more than one baseline abnormality (top two curves) or one or less abnormality (lower two curves). Shown are Kaplan-Meier curves of cancer incidence rates in patients who are NSAID nonusers (former or never users, red curves) or NSAID users (current or user during follow-up, black curves).

**Figure 2:**
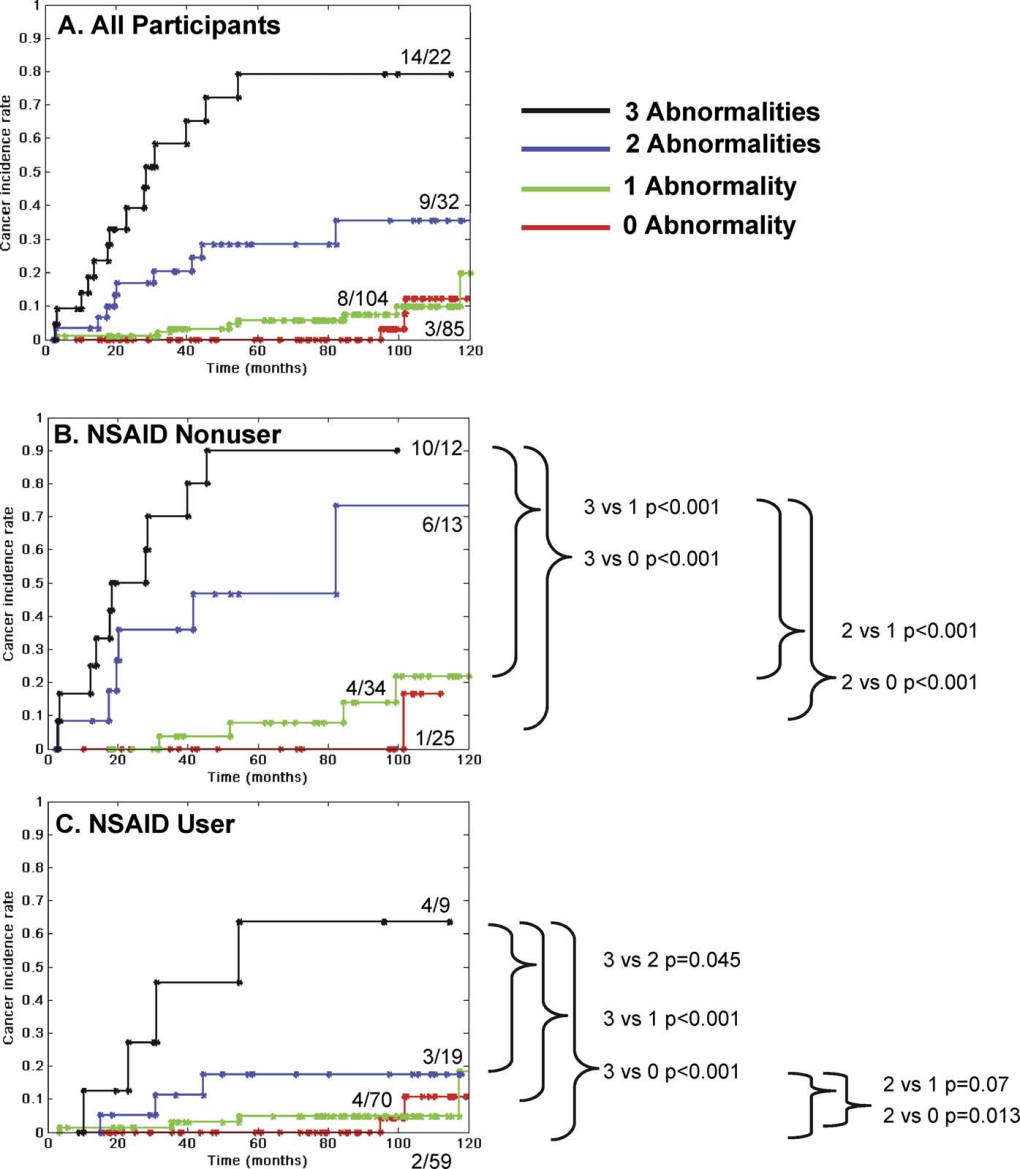
Cumulative EA Incidence with Combinations of Abnormalities (17p LOH, DNA Content Abnormality, 9p LOH) in NSAID Nonusers and NSAID Users Cancer incidence rates are shown for participants with no selected abnormalities (17p LOH, DNA content abnormalities [aneuploidy and/or tetraploidy], or 9p LOH) at baseline (red), any one abnormality (green), any combination of two abnormalities (blue), or all three abnormalities (black). (A) All participants. When comparing NSAID nonusers (B) and NSAID users (C) there is a strong significant trend toward EA risk reduction in the NSAID users group for all abnormality combinations (Mantel-Haenszel test *p* = 0.01).

We sought to evaluate the extent to which specific tissue-based, mechanistically derived molecular markers, both alone and in combination, predict risk of progression to EA in patients with BE. The analytic approach is summarized as follows. (1) Univariate Cox proportional hazard analyses (not adjusted for host variables or other markers) were used to determine the EA risk of patients with each marker, measured at baseline at 2-, 6-, and 10-y follow-up time points (Table 2). (2) Stepwise multivariate Cox regression was used to select, from among all of the available molecular markers, a subset which independently contributes to EA risk prediction (Table 3). (3) Cumulative EA incidence and relative risk (RR) of progression to EA at 10 y were calculated for patients with different numbers of the selected markers at baseline relative to patients with no abnormalities (Table 4). To determine the contribution of known or suspected nongenetic host factors to EA risk prediction, we incorporated these host factors together with all molecular markers in a multivariate Cox model. Only NSAID use showed significant independent prediction in combination with molecular markers in this multivariate model. The predictive ability of the selected molecular markers was evaluated among NSAID users and nonusers (Figures 1 and 2).

**Table 2:**
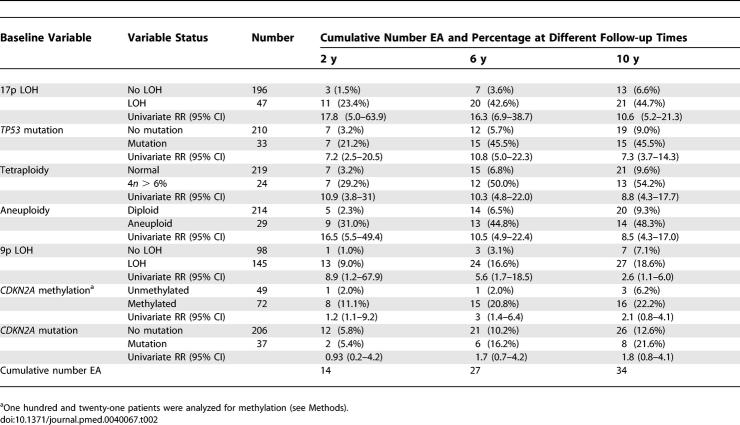
Univariate Analysis of RR for EA during Follow-up

**Table 3:**
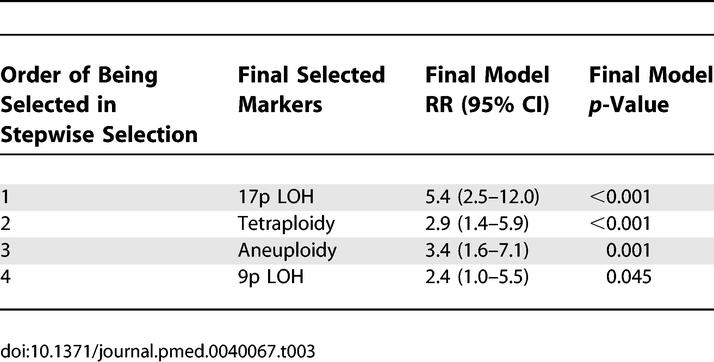
Stepwise Selection for Molecular Markers and RR for EA of Final Selected Markers

**Table 4:**
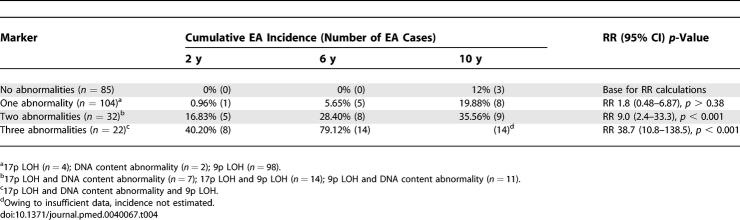
Cumulative EA Incidence and RR of Different Baseline Abnormality Combinations

Two hundred and forty-three patients had informative data for all of the molecular markers (17p LOH, *TP53* mutation, tetraploidy, aneuploidy, 9p LOH, and *CDKN2A* mutation). Except for *CDKN2A* methylation, all markers in the analysis were binary variables coded as 0 (no), 1 (yes) for each participant. *CDKN2A* methylation status was coded as yes, no, or no data in the statistical model because only 121 patients had sufficient quantity of DNA to be evaluated for this marker. In the Cox regression models for molecular marker selection, the statistical significance levels used in the initial process of stepwise selection for variable entry and removing were 0.25 and 0.15, respectively. Based on initial selection results, *p* = 0.05 was used as the significance threshold for the final selection (second Cox model). Association relationship among binary markers was also assessed using coefficient Φ. The Kaplan-Meier method was used to estimate the probability of EA in different stratified groups. The Gehan-Wilcoxon test was used to test differences in the cancer incidence curves. The Grambsch-Therneau test was used to examine the significance of a change in RR ratio over time for all markers. We adjusted *p*-values for multiple comparisons in Figure 2 using the Benjamini and Hochberg method [57]. All of the analyses were performed with Statistical Analysis System (SAS) software Version 9.0; (SAS Institute, http://www.sas.com).

## Results

### Univariate Analysis of LOH, Mutation, Methylation, Tetraploidy, and Aneuploidy for EA Risk Prediction

The cohort characteristics for participants used for molecular and DNA content analysis are shown in Table 1. During the follow-up period of this study, 34 participants developed EA. The number of participants with each genetic abnormality at baseline and the cumulative number who developed cancer at 2, 6, and 10 y are shown in Table 2. The risk of EA during follow-up in patients with each molecular and DNA content abnormality was assessed with univariate analysis using Cox regression models (2, 6, and 10 y presented to show general trend, Table 2). The RRs presented in Table 2 were not adjusted for host variables, but the significance of the results does not change when such adjustments are performed. At 10 y, each molecular and DNA content abnormality, when analyzed alone in a patient at baseline, made a significant contribution to prediction of EA risk, with the exception of *CDKN2A* mutation (10-y RR = 1.8; 95% CI 0.8–4.1, *p* = 0.13) and *CDKN2A* methylation (RR = 2.1; 95% CI 0.8–4.1, *p* = 0.09). For example, the univariate RRs ranged from a high for 17p LOH (10-y RR = 10.6; 95% CI 5.2–21.3, *p* < 0.001) to that for 9p LOH (10-y RR = 2.6; 95% CI 1.1–6.0, *p* = 0.03). Although RRs at intermediate time points may be unstable owing to small numbers, 9p LOH had a higher RR at early follow-up intervals. The RR in univariate analysis for 9p LOH was 8.9 (95% CI 1.2–67.9, *p* = 0.04) at 2-y follow-up, but decreased to RR = 2.6 by 10 y. With the current dataset, 9p LOH was the only abnormality that showed a statistically significant change in RR over time (nonproportional hazard over time, *p* = 0.004, Grambsch-Therneau test for global trend at 10 y).

### Comprehensive Analysis of Relationships among the Multiple Molecular Abnormalities for EA Risk Prediction

To determine a set of biomarkers that are independent predictors of EA risk, baseline molecular markers were evaluated using multivariate Cox models with backward and forward stepwise selection. Starting with all molecular abnormalities for selection, 17p LOH was the most significant abnormality selected by the statistical model, and remained a strong predictor when accounting for *TP53* mutation status in a step-wise analysis process. Although *TP53* mutation was highly significant in univariate analysis, it became nonsignificant for EA risk prediction when 17p LOH was included in the model. Tetraploidy was the next selected marker, followed by aneuploidy. Finally, 9p LOH improved the model for risk prediction significantly in combination with 17p LOH and DNA content abnormalities (likelihood ratio test *p* = 0.03). *CDKN2A* methylation provided marginal additional risk but was not statistically significant in the multivariate model (*p* = 0.28). *CDKN2A* mutation did not provide significant additional contribution to EA risk prediction. The final selected molecular markers that independently contributed to EA risk prediction included 17p LOH, tetraploidy, aneuploidy, and 9p LOH (Table 3). To further evaluate the associations among the markers, we calculated the association coefficient (Φ) for all binary markers. *TP53* mutation showed strong association with 17p LOH (Φ = 0.63) and aneuploidy (Φ = 0.56). 17p LOH is also strongly associated with aneuploidy (Φ = 0.56). No other strong associations were observed by this method. Although *TP53* mutation was significantly related to EA risk in univariate analysis, it was strongly associated with 17p LOH and aneuploidy, which may explain why it was not retained in the model selection. The adjusted RRs for future EA at 10 y, using the final selected model with 17p LOH, tetraploidy, aneuploidy, and 9p LOH, were RR = 5.4 (95% CI 2.5–12.0), RR = 2.9 (95% CI 1.4–5.9), RR = 3.4 (95% CI 1.6–7.1), and RR = 2.4 (95% CI 1.0–5.5), respectively (Table 3).

### Effect of Combinations of Selected Panel Abnormalities on Cumulative EA Incidence

Each study participant had the potential to have any combination of selected panel abnormalities at baseline. Stratification by all possible combinations of the selected abnormalities was limited by an inadequate number of patients for analysis in some strata, and we therefore grouped participants based on the number of abnormalities at baseline. Table 4 lists the cumulative EA incidence at 2, 6, and 10 y, and the RR at maximum follow-up (10 y) for future development of EA for participants with no panel abnormalities, a single abnormality (either 17p LOH, any DNA content abnormality, or 9p LOH), any two selected abnormalities, or all three selected abnormalities at baseline. Tetraploidy and aneuploidy are two measures of DNA content and were treated as a single variable in this section (Figures 1 and 2; Table 4). Study participants with no baseline abnormalities (85/243) remained cancer free to almost 8 y (95 mo) and had a relatively low 10-y cumulative cancer incidence of 12%. In participants with only a single baseline abnormality (104/243), the 6-y EA incidence was 5.65%, with an overall 10-y EA incidence of 19.88%. It is of note that 9p LOH was the single abnormality in 98/104 of those participants, indicating that it was rare for 17p LOH or a DNA content abnormality to be detected alone. Participants with two abnormalities detected at baseline (32/243) had increasing cumulative EA incidence of 16.83%, 28.4%, and 35.56% at 2, 6, and 10 y, respectively, showing a significantly higher EA risk than for patients with no abnormalities at baseline (RR = 9.0; 95% CI 2.4–33.3, *p* < 0.001). Participants (22/243) with all three abnormalities (17p LOH, DNA content abnormality, and 9p LOH) had EA incidence rates of 40.2% and 79.12% at 2 and 6 y, respectively. Owing to sample size, the EA incidence rate at the 10-y time point was not estimated. These patients had the highest risk of cancer relative to those with a no baseline abnormalities (RR = 38.7; 95% CI 10.8–135.5, *p* < 0.001). Of these cancer cases, 80% occurred within 36 mo, and all cancers that developed in patients with three baseline abnormalities did so within a 55-mo follow-up period.

### Association of NSAID Use with Reduction of EA Risk in Participants with 17p LOH, DNA Content Tetraploidy and Aneuploidy, and 9p LOH

Multiple nongenetic host factors and demographic variables have been previously suggested as potential modifiers of EA progression including age, gender, waist-to-hip ratio, smoking status, segment length, and NSAID use [24,58–67]. To determine the host variables that provide independent prediction for EA when combined with molecular markers, we incorporated these nongenetic host factors with all molecular markers into a new Cox model for variable selection. The selected independent variables were 17p LOH, DNA content abnormalities (tetraploidy and aneuploidy), 9p LOH, and NSAID use. In combination with the selected molecular abnormalities, NSAID use was associated with a statistically significant reduction of EA risk (*p* < 0.001). Thus, NSAID use was evaluated as a potential modulator of cancer risk in combination with selected genetic abnormalities. For a given selected molecular marker, Kaplan-Meier analysis showed that NSAID use during follow-up provided a significant association with protection against development of EA, relative to participants who were NSAID nonusers (former or never) (Figure 1). This protective effect was significant in patients with baseline 17p LOH (*p* = 0.004, Gehan-Wilcoxon test), DNA content abnormalities (tetraploidy and/or aneuploidy) (*p* = 0.01), and 9p LOH (*p* <0.001) (Figure 1A–C). For patients with more than one panel abnormality at baseline, NSAID use was associated with a significant reduction in EA incidence (*p* = 0.001): NSAID nonusers had an observed cumulative EA risk of 68%, compared to 30% for NSAID users at 6 y, and 79% compared to 30% for NSAID users at 10 y (Figure 1D). In participants with either no abnormalities or one abnormality at baseline (predominantly 9p LOH, Table 4), cancer risk was low, and the protective effect of NSAIDs for future EA was not significant (*p* = 0.28 at 10-y follow-up).

### EA Incidence in NSAID Nonusers and NSAID Users Stratified by Combinations of Baseline Abnormalities

Cancer incidence of all participants based on the overall number of select panel abnormalities at baseline is shown in Kaplan-Meier curves (Figure 2A). When comparing NSAID nonusers (Figure 2B, *n* = 84) and NSAID users (Figure 2C, *n* = 157) within all categories (zero, one, two, or three abnormalities at baseline), there was a strong significant trend toward EA risk reduction in the NSAID user's group (Mantel-Haenszel test *p* = 0.01). NSAID nonusers with two or three abnormalities at baseline had a highly significant increased EA incidence compared to NSAID nonusers with zero or one lesion (two versus zero *p* < 0.001; two versus one *p* <0.001; three versus zero *p* < 0.001; three versus one *p* < 0.001; adjusted for multiple comparisons with Benjamini and Hochberg method [57] [Figure 2B]). All other comparisons for NSAID nonusers, for example between participants with two versus three abnormalities, were nonsignificant. Participants who used NSAIDs regularly (Figure 2C) and had all three abnormalities at baseline had a significantly increased cumulative EA incidence compared to NSAID users with zero, one, and two abnormalities (*p* < 0.001, *p* < 0.001, and *p* = 0.045, respectively). NSAID users with two baseline abnormalities had a significant or marginally significant increased cancer incidence relative to NSAID users with zero or one abnormality (*p* = 0.013 and *p* = 0.07, respectively).

## Discussion

This investigation reports the results of a prospective cohort study of mechanistic-based genetic abnormalities evaluated as predictors of EA and demonstrates the modulating effect of NSAIDs on EA risk in patients with BE. We hypothesized that a panel of somatic genetic abnormalities involving *TP53, CDKN2A,* and DNA content could improve prediction of progression to EA and that NSAID use may modulate EA risk. In this longitudinal study spanning more than a decade, we showed that a combination of 17p LOH, 9p LOH, and DNA content tetraploidy and aneuploidy provide significant, independent EA risk prediction. NSAID use is associated with reduction of EA risk, and the protective effect was highly significant for patients who have multiple high-risk molecular abnormalities at baseline. These analyses include 34 EA endpoints, which is second only to our previous 15-y report of histology and flow cytometry (42 EAs) and substantially larger than most other longitudinal studies of biomarkers in BE from other centers, which have typically reported 12 or fewer incident cancers [41,42,68,69]. This prospective study has been conducted in a single center with a high-risk cohort; studies in other centers will be required to determine whether our results can be generalized to other patient populations and to validate the results for clinical application. Our results are consistent, however, with previous longitudinal studies of single biomarkers from other centers, including *TP53* abnormalities and flow cytometry [41,42,70]. To our knowledge, no previous studies in patients with BE or any other human premalignant condition have prospectively evaluated the contributions of *TP53* and *CDKN2A* gene inactivation (methylation, mutation, and LOH) and DNA content abnormalities in combination with candidate interventions to assess their potential utility as biomarkers for future cancer risk and cancer prevention.

The mechanisms by which *TP53* and *CDKN2A* regulate the cell cycle under normal and abnormal conditions have been investigated extensively in elegant molecular studies in vitro and in model organisms. Perturbations of these genes and the pathways in which they act have profound, mechanistic associations with human cancer based upon evidence accumulated in numerous laboratories [1,71–75]. In BE, neoplastic progression is characterized by clonal evolution in which genetic instability generates variants on which natural selection acts, resulting in waves of clonal expansion, generation of new variants, and further selection [76,77]. *TP53* abnormalities typically arise in clones with *CDKN2A* abnormalities [78], creating a condition permissive for clonal variants, including tetraploid and aneuploid populations, to survive and expand [79]. Thus, the abnormalities in this biomarker panel assess viable clones that undergo expansion *(CDKN2A)* and survive chromosomal instability (*TP53,* DNA content). Assessment of multiple stages of clonal evolution may be the basis for improved risk stratification compared to single biomarkers.

We simultaneously measured DNA content abnormalities (tetraploidy and aneuploidy), inactivation of *TP53* (mutation and LOH), and inactivation of *CDKN2A* (mutation, methylation, and LOH), all of which have been shown to be mechanistically related to neoplastic progression in BE [28,29,34,80–82]. *TP53* abnormalities have been shown in numerous studies to be predictive of EA [41,70,83–85]. In the present study, *TP53* mutations were strongly associated with 17p LOH and aneuploidy and were not selected in the multivariate analysis. Selection of LOH over methylation or mutation in the Cox model could occur because LOH is a common manifestation of the chromosomal instability that is characteristic of neoplastic progression in BE [29]. LOH could also be selected as the “second hit” for inactivating *TP53* and *CDKN2A.* Alternatively, LOH events often span large chromosomal regions that could include other genes such as *HIC1* (17p13.3) [86] and *p14^ARF^* (9p21) [72,87] that may confer additional selective advantages over mutational or methylation events that affect only *TP53* and *CDKN2A.* Recently, Maley et al. reported that clonal diversity measures derived from evolutionary biology retained significant independent EA risk prediction with 17p *(TP53)* LOH and abnormal ploidy, but 9p LOH became nonsignificant when incorporating evolutionary variables [88]. The nonproportional hazard varying with time that we found for 9p LOH in the present study may reflect the genetic background of the *CDKN2A* clone. For example, expansion of a *CDKN2A* abnormal clone that is otherwise genetically stable may homogenize the neoplasm, minimizing diversity on which natural selection might act to promote progression [76,77]. In contrast, expansion of a *CDKN2A* abnormal clone predisposed to genetic instability through either environmental or somatic genetic factors would result in increased diversity that could promote progression.

Although this study had a large number of EA endpoints for published studies of BE, the number of cancers was relatively small compared to studies in breast and colon, for example. Therefore, estimates of RR over time showing the 9p LOH nonproportional hazard over time need to be further investigated in future studies. In addition, RR for EA presented in Table 2 at intermediate follow-up times should be treated with caution. We do not recommend that clinicians manage patients on these data alone. Within the BE research field, there is a paucity of data concerning progression to EA in patients with different molecular abnormalities. We present the intermediate time points to inform design of future intervention and multicenter studies, while maintaining statistical rigor in our analyses.

Identification of host genetic factors, including inherited, highly penetrant mutations in cancer susceptibility for hereditary breast cancer *(BRCA1, BRCA2),* familial polyposis coli *(APC),* and those predisposing to hereditary non-polyposis colon cancer, among others, in combination with knowledge of environmental factors, have the potential to reduce cancer morbidity and mortality by early detection and prevention [5,6,9,10,89–91]. In contrast to inherited mutations in relatively uncommon susceptibility genes, less is known concerning temporal progression of somatic genetic abnormalities in more prevalent sporadic premalignant conditions. Genetic progression models have been proposed for many types of cancers based largely on cross-sectional data [11,13,14,92–104]. *TP53, CDKN2A,* and DNA content (tetraploidy and aneuploidy) abnormalities are among the most common abnormalities in cancers and premalignant conditions affecting multiple organs, including head and neck, lung, breast, bladder, and pancreas, among others [1,71–73]. Few data exist as to their ability to predict future cancer or how these lesions can be modulated by chemoprevention efforts. Advances have been made in cancer risk prediction for patients with oral premalignant lesions in multiple retrospective, longitudinal studies [13–16]. Lee et al. combined multiple biomarkers and patient characteristics in Cox regression analysis and found that chromosome polyploidy, together with high *p53* expression, LOH, and histology was the best predictor of cancer risk in a 10-y study of 70 patients with 22 cancer outcomes [13]. Thus, genetic progression models may be a rich source of hypotheses for retrospective longitudinal and prospective biomarker validation studies.

In experimental model systems and observational studies, aspirin and other NSAIDs have been reported to inhibit cyclo-oxygenase 2, increase apoptosis, decrease inflammation, decrease proliferation, and inhibit angiogenesis [4,26,27,105–111]. It has recently been shown that the absolute size of aneuploid clones and clones with *TP53* lesions is a risk factor for progression to EA [79]. NSAIDs may act by reducing clone size through increasing apoptosis and decreasing angiogenesis and proliferation. By increasing apoptosis, NSAIDs may decrease the generation of viable clones, thus decreasing diversity and limiting the pool of genetic variants on which natural selection may act. In addition, NSAIDs function to reduce inflammation, which may in turn reduce the mutation rate in evolving clones and decrease the number of cellular variants. The cohort analyzed in the present report is a subset of that described in Vaughan et al. [25]. Of the molecular cohort described in the present study, 65% were current NSAID users—including use in follow-up—and this proportion is comparable with the 63% regular NSAID users (including use in follow-up) in the total cohort. About half of the users took aspirin for reasons concerning cardiovascular health, and the vast majority of the other users primarily took ibuprofen for pain relief. In a previous paper, we reported a strong protective association for EA with current NSAID use (at baseline or during follow-up), a rather rapid diminution of the association among former users, and no evidence of a stronger association with increasing frequency and/or duration of use. Furthermore, we did not find significant differences in risk of EA or aneuploidy according to type of NSAID. Thus for the present report, our patients were classified simply as NSAID user or nonuser (former or never) based on use at least once per week for ≥6 mo any time during follow-up, regardless of daily frequency, duration, or type. By focusing on the salient results from the previous analyses of a larger dataset regarding NSAIDs, we avoided multiple comparisons and could focus on the relationship of NSAID use with the status of molecular abnormalities.

Our results show a protective association between NSAID use and progression to EA in all participants, and particularly among those with multiple somatic genetic abnormalities. The vast majority of patients in this cohort had gastroesophageal reflux disease and were undergoing therapy, predominately using proton pump–inhibitors, to reduce reflux symptoms. It is unclear as to how the frequency or severity of symptoms may affect NSAID use by participants. However, to our knowledge, symptoms from reflux are not associated with intermediate endpoints or cancer in BE, so it is unlikely that symptoms could explain the association of NSAID use with reduced risk of EA. None of the patients had endoscopically visible concomitant conditions of the stomach, such as ulcers, at the baseline endoscopy that could have conceivably altered NSAID use. Given the experimental evidence provided in model systems and in previous studies, we propose that NSAIDs act within the BE tissue to modulate progression to EA. Our study is early translational research, as defined by the National Cancer Institute Translational Research Working Group (http://www.cancer.gov/aboutnci/trwg/presentations). These results advance our understanding of the molecular mechanisms of neoplastic progression as well as the mechanisms by which aspirin and other NSAIDs may prevent cancer. As such, these results are consistent with the National Institutes of Health goals of prevention, prediction, and personalized medicine. Several other types of research will be essential for these results to reach the clinic: multicenter randomized trials with a mechanistic focus to determine the effects of aspirin or other NSAIDs on progression to EA and intermediate endpoints (high-risk biomarkers), health services research to evaluate the effect of the interventions as well as the cost effectiveness of biomarkers that reduce frequencies of endoscopy and numbers of biopsies, and establishment of reimbursement mechanisms to support dissemination and adoption (National Cancer Institute's translational continuum; http://www.cancer.gov/trwg/TRWG-definition-and-TR-continuum).

Our study builds on a body of observational research, a recent meta-analysis, and supportive data in other cancers, most notably colon cancer where several clinical trials have reported a protective association of aspirin and other NSAIDs, although some findings have been inconsistent [9,10,21–25,89–91,112–116]. The present study extends these findings by evaluating the association of NSAID use with somatic genetic events that define progression in BE and demonstrates the benefits of NSAID use in patients at high risk of progressing to EA. Our results are consistent with previous results showing a protective effect on the part of NSAIDs in patients with HGD [25], and with computer models indicating that high-risk patients are the most likely to benefit from NSAID intervention [117]. The incidence of EA is sufficiently low in BE that designing statistically rigorous, adequately powered prevention studies with cancer as an endpoint is not feasible for most research cohorts. Thus, knowledge of the interaction of NSAIDs and somatic genetic abnormalities will help define entry criteria for randomized intervention trials with a cancer endpoint. The combination of a somatic genetic biomarker panel that identifies those patients with BE who are at high risk of progression to EA, combined with an inexpensive, widely available, and relatively safe means of preventing neoplastic progression in such high-risk patients, could have significant public health and economic benefits.

## Supporting Information

### Accession Numbers

The GenBank (http://www.ncbi.nlm.nih.gov/Genbank) accession numbers for the genes discussed in this paper are *TP53 (p53)* (7157), *CDKN2A (p16, p16-INK4a)/p14^ARF^* (1029), and *HIC1* (3090).

## Abbreviations

BE: Barrett's esophagus
CI: confidence interval
EA: esophageal adenocarcinoma
HGD: high-grade dysplasia
LOH: loss of heterozygosity
NSAID: nonsteroidal anti-inflammatory drug
RR: relative risk
